# Combined Nanoparticle-Based Delivery of Estrogens and Raloxifen in Postmenopausal Osteoporosis

**DOI:** 10.3390/nano16030180

**Published:** 2026-01-28

**Authors:** Agnieszka Włodarczyk, Patrycja Dolibog

**Affiliations:** Department of Medical Biophysics, Faculty of Medical Sciences in Katowice, Medical University of Silesia, 40-055 Katowice, Silesia, Poland

**Keywords:** postmenopausal osteoporosis, estradiol, raloxifene, nanoparticles, side effects

## Abstract

Osteoporosis (OP) is a common chronic disease that significantly increases the risk of bone fractures. Pharmacotherapy uses, among others, 17beta-estradiol (E2), which has been replaced in recent years by raloxifene hydrochloride (RLX). The need for long-term, high-dose therapy with these drugs is associated with serious adverse effects. The aim of this review is to analyze the current state of knowledge over the last 5 years (2020–2025) regarding the use of nanoparticles (NPs) in the delivery of E2 and RLX, with particular emphasis on their impact on bioavailability, pharmacokinetic profile, reduction in adverse effects, and improvement in the effectiveness of postmenopausal osteoporosis therapy. Preclinical studies show that combining E2 or RLX with various types of NPs reduces cytotoxicity, improves pharmacokinetic parameters, and enhances the therapeutic effects of drugs used in postmenopausal osteoporosis. These effects are mainly attributed to improved pharmacokinetics and controlled drug release, rather than confirmed active tissue targeting. However, these findings are based on preclinical models and require further validation in clinical studies. The analysis concludes that while NP systems significantly enhance the pharmacokinetic profile and safety of E2 and RLX in preclinical models, claims of true bone-specific targeting remain largely unsubstantiated, highlighting a key area for future research.

## 1. Introduction

Osteoporosis (OP) is a common chronic disease that significantly increases the risk of bone fractures. Osteoporotic fractures contribute to increased mortality and a decreased quality of life. For older women, whose disease develops as a result of estrogen deficiency, osteoporosis has been recognized as a major public health problem. Therefore, effective fracture prevention measures are crucial [1].

The primary cause of postmenopausal osteoporosis is a deficiency of estrogen, thyroid hormone, and calcitonin, which results in increased osteoclast differentiation and activation. This leads to accelerated bone resorption, which exceeds the rate of bone regeneration, leading to rapid bone mass loss. This results in reduced bone mineral density, deterioration of bone microarchitecture, reduced mechanical strength, and an increased risk of fractures due to bone fragility [2,3]. Estrogens play a key role in maintaining bone mineral mass and stabilizing its metabolism by inhibiting osteoclast resorption and stimulating osteoblast osteogenesis [4,5].

The mechanisms of postmenopausal osteoporosis resulting from decreased estrogen levels include the following:

1. Increased production of proinflammatory cytokines such as interleukin 1 (IL-1) and tumor necrosis factor (TNF), which are potent stimulators of bone resorption and well-known suppressors of bone formation. They also stimulate osteoblasts to produce interleukin-6 (IL-6), which activates osteoclast precursors.
2. Reduced activation of the Fas/FasL (CD95/APO-1) pathway, which normally regulates osteoclast apoptosis. Estrogen supports this mechanism, so its deficiency prolongs the osteoclast lifespan, enhancing bone resorption.
3. Inhibition of osteoprotegerin (OPG) expression in stromal cells and osteoblasts, a protein secreted by osteoblasts and bone marrow stromal cells that acts as a “trap” for receptor activator of nuclear factor kappa-B ligand (RANKL), preventing its binding to the RANK receptor on osteoclast precursors. Decreased OPG levels lead to excessive osteoclast activation and increased resorption.
4. Both direct and indirect intensification of the RANK/RANKL pathway. RANKL binds to the RANK receptor on osteoclast precursors, activating their differentiation and function. This is a key pathway in the regulation of bone turnover, whose dysregulation accelerates bone loss [6].

An important feature of estrogens is also their antioxidant effect on free radicals, the concentration of which increases as a result of aging. This also contributes to increased bone resorption during menopause [7].

It is worth noting that, in addition to pharmacological therapy, recent reviews have highlighted the importance of non-pharmacological strategies for fracture prevention in older adults, including resistance and balance exercises, fall prevention, calcium, vitamin D and protein supplementation, and vibration therapy. Integrating such interventions into care systems, such as Fracture Liaison Services, may further enhance fracture prevention and improve clinical outcomes. Furthermore, recent reviews have highlighted the importance of post-translational modifications (PTMs) in bone remodeling, which may represent a new therapeutic direction, although clinical evidence is still limited [8,9].

There are three categories of treatment for postmenopausal osteoporosis. The first is antiresorptive therapy, which includes bisphosphonates, denosumab, selective estrogen receptor modulators (SERMs), and estrogen. These medications reduce bone resorption and slow bone loss. Among the commonly used antiresorptive drugs, calcitonin is included. However, in recent years, concerns regarding its long-term safety have emerged, with some reports suggesting a potential association with malignancy [10].

The second category is anabolic therapy, represented by teriparatide and abaloparatide, which are parathyroid hormone (PTH) analogs that stimulate bone formation. The third treatment option is romosozumab, a dual-action drug that both increases bone formation and decreases bone resorption [11].

In clinical practice, osteoporosis treatment often relies on antiresorptive medications due to reimbursement criteria and guidelines. Bisphosphonates require high therapeutic doses because of low bioavailability, which can cause adverse effects such as esophagitis, jaw osteonecrosis, bone pain, pulmonary edema, and fatigue [12,13]. Denosumab treatment may be associated with hypocalcemia, sensory abnormalities, muscle spasms, stiffness, and prolonged QT interval [14]. Some patients discontinue denosumab for various reasons, including financial ones [15].

17beta-Estradiol (E2) is used in the treatment of osteoporosis, but its use is associated with serious side effects, which are intensified by the need to use high doses [6,16,17]. Due to the risk of adverse effects associated with estradiol therapy, research is underway to solve these concerns. A central premise of this review is that nanoparticle-based delivery systems can improve estrogen pharmacokinetics and overall drug distribution; however, despite these advantages, true tissue-specific targeting has not yet been conclusively demonstrated. Such systems may enhance therapeutic efficacy and reduce systemic exposure by enabling controlled release and lower dosing [3,18,19,20]. Another solution to the side effects of E2 therapy is to replace it with estrogen-like drugs that selectively target tissues such as the heart and bones, but not the breast tissue. These drugs are called selective estrogen receptor modulators (SERMs) [21,22]. An example of a drug with this effect is raloxifene hydrochloride (RLX, RAL). It is a second-generation drug approved by the U.S. Food and Drug Administration (FDA) in 1997 for use in increasing bone density and reducing the risk of postmenopausal fractures. RLX is considered one of the safest drugs used in the prevention and treatment of osteoporosis, when used long-term [23].

Its extensive clinical evidence, long-term safety profile, and additional benefit in reducing the risk of invasive breast cancer in postmenopausal women support its selection as the central focus of this review.

Raloxifene belongs to the benzothiophene class and exhibits estrogen agonist activity in the context of bone and lipid metabolism. At the same time, it acts antagonistically in tissues such as the mammary gland and endometrium. Clinical trials have confirmed its effectiveness in both preventing bone loss and reducing the risk of breast cancer [24]. RLX is characterized by low aqueous solubility. Furthermore, like E2, it undergoes extensive intestinal glucuronidation before gastrointestinal passage and phase II metabolism in the liver (first-pass metabolism). Due to these limitations, the oral bioavailability of RLX is lower, at only 2%. These limitations mean that this drug does not solve the problem of adverse effects, but merely modifies their profile [25,26,27,28]. Due to its poor solubility, no injectable form of RLX has been developed [26]. The above-mentioned features have minimized the therapeutic use of RLX; therefore, there is a need to develop better and more effective dosage forms to enhance its clinical efficacy in patients [23]. The use of nanoparticles (NPs) in medicine offers a number of advantages over conventional drug forms. The most important advantages include improved bioavailability of active substances; greater therapeutic efficacy with lower doses and less frequent administration; the ability to improve drug distribution and control release, although true tissue-specific targeting remains unconfirmed; extended shelf life; and improved patient tolerance and comfort [27]. They also demonstrate the ability to penetrate smaller capillaries and be absorbed by cells, supporting enhanced drug penetration and cellular uptake, rather than confirmed precise targeting [19]. Due to their physical and chemical properties, they can be used to create drug delivery systems (DDSs). In recent years, interest in using NPs as DDSs for the treatment of bone diseases has increased. The therapeutic agent would be released into bone tissue via the drug carrier, promoting bone formation and/or reducing bone resorption. DDSs thus optimize drug dosing, protect against biodegradation, and reduce exposure of non-target cells to the drug [17].

The aim of this review is to analyze the current state of knowledge in the last 5 years (2020–2025) on the use of nanoparticles in the delivery of E2 and RLX, with particular emphasis on their impact on bioavailability, pharmacokinetic profile, reduction in side effects, and potential improvement in therapeutic effectiveness in the treatment of postmenopausal osteoporosis. This work is a narrative review summarizing preclinical evidence. The text was revised to more clearly highlight the significance of the in vivo findings and their possible clinical implications.

## 2. Materials and Methods

This article is a narrative review; therefore, no original experimental procedures are included.

### 2.1. Inclusion Criteria

Articles related to research on the use of E2 and RLX with nanoparticles in the treatment of osteoporosis from the last 5 years (2020–2025) from the PUBMED and Google Scholar databases were used to generate this work. Only original, experimental, and preclinical articles on animals and cell lines related to bone metabolism, written in English, were included.

### 2.2. Exclusion Criteria

Papers regarding nanoparticles in combination with estradiol and RLX for indications other than osteoporosis and those not containing data on bone were excluded. Review articles were also excluded.

## 3. Results

### 3.1. Treatment of Postmenopausal Osteoporosis

Treatment for bone loss includes the use of hormone replacement therapy (HRT), but like any approved treatment, it has certain side effects. Until recently, 17β-estradiol (E2) was widely used in the prevention and treatment of postmenopausal osteoporosis. However, its oral administration is associated with extensive first-pass hepatic metabolism, which significantly limits bioavailability. Consequently, high doses are required to achieve a therapeutic effect, which increases the risk of adverse effects [29]. Due to this, the need for a long treatment period, and the fact that estrogens also reach tissues other than bone, HRT in menopausal women increases the risk of stroke and cardiovascular disease, including venous thromboembolism; multiplies the risks of congestive heart disease (CHD), breast cancer, endometrial cancer, and weight gain; and could even cause cholecystitis (Table 1) [6,16,17]. This is because systemically administered drugs circulate throughout the body after being absorbed into the bloodstream. They show limited penetration into bone tissue and are rapidly cleared from the body. Furthermore, drugs penetrate bone less than other tissues because bone has less vascularization than other organs, such as the brain, liver, or kidneys. As a result, they are often administered in high doses, which can cause systemic toxicity [17]. Considering these adverse effects, systemic estradiol therapy is often not recommended for elderly women due to an unfavorable risk–benefit profile [19]. Therefore, E2 therapy has been replaced in recent years by RLX. Unfortunately, to be effective, it must be administered at a dose of 60 mg once a day continuously for more than two years [27], which may be associated with adverse events such as hot flashes, nausea, deep vein thrombosis, stroke, leg cramps, swelling, and cold symptoms [27]. Furthermore, recent studies show that eye conditions such as cataracts and macular degeneration, as well as gynecological problems like uterine polyps and hemorrhage, have also been reported. Furthermore, the analysis confirmed that pulmonary embolism and deep vein thrombosis are the two most common thromboembolic adverse events [28].

### 3.2. Nanoparticles

The definition introduced by the European Commission states that nanoparticles are materials in which at least half of the particles are equal to or less than 100 nm. They are structures with unique properties, characterized by a high active-surface-area-to-volume ratio [30]. They can be synthesized from various organic materials, such as polymers, hydroxyapatite, and even lipids and proteins. They can also be inorganic, for example, metal- or metal oxide-based particles. These inorganic nanoparticles can additionally exhibit magnetic properties, for example, superparamagnetic iron oxide (Fe_3_O_4_) nanoparticles [31,32]. To date, nanoparticles have been extensively studied for medical applications. Their physical and chemical properties enable their use in diagnostics, therapies, drug delivery, and disease imaging [33].

**Figure 1:**
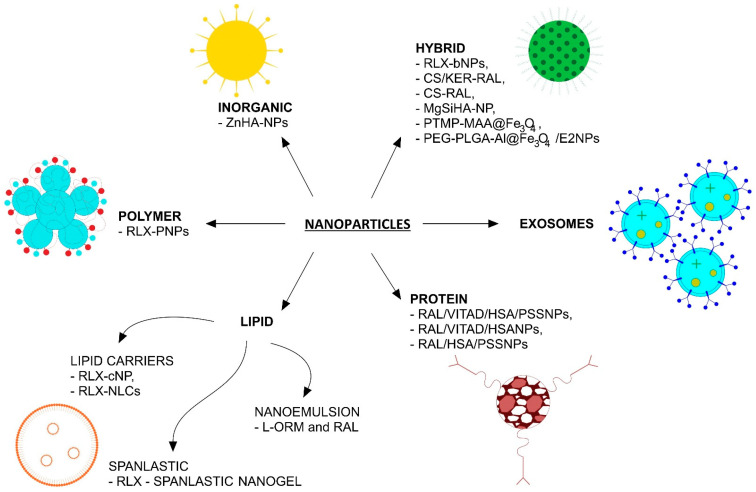
Summary of nanoparticle systems reported in the last five years for estradiol (E2) and relaxin (RLX) delivery in osteoporosis-related preclinical studies. The schematic includes commonly applied in vitro safety evaluations, such as MTT assays and hemolysis tests, where available. CS—chondroitin sulfate; KER—keratin; HSA—human serum albumin; PLGA—poly(lactic-glycolic acid); PEG—polyethylene glycol; AL—alendronate; PTMP-PMAA—the multifunctional water-soluble polymer; PSS—sodium polystyrene sulfonate; VitaD—vitamin D_3_; L-ORM—levomeloxifene; ZnHA—nano zinc hydroxyapatite; Mg—magnesium; Si—silicon; bNPs—polymer/lipid hybrid nanoparticles; cNPs—lipid nanoparticles.

A promising area of research exploring the use of nanoparticles in osteoporosis treatment is the use of hydroxyapatite (HA) nanoparticles. As a natural mineral matrix of human bones and teeth, it exhibits high osteoconductive properties and biocompatibility. Using HA as a nanocarrier promotes bone mass deposition and bone tissue growth [34,35]. The authors of the cited study suggest that HA-based nanocarriers can enhance the preferential accumulation of biomolecules in bone tissue due to their affinity for the mineralized matrix, rather than achieving precise tissue-specific targeting [36]. In addition to these promising results regarding nanohydroxyapatite, in recent years, new classes of nanoparticles have also emerged which, when conjugated with E2 or RLX, show therapeutic potential in the treatment of postmenopausal osteoporosis. Figure 1 shows the nanoparticles used in preclinical studies on E2 and RLX delivery for the treatment of postmenopausal osteoporosis in the last 5 years (2020–2025).

Thanks to their properties, nanoparticles can encapsulate drugs and improve their biocompatibility. Once encapsulated, drugs can be protected, carried within the organism, and released in a more controlled manner. Such systems may enable lower dosing and reduce systemic exposure, potentially decreasing side effects and enhancing therapeutic outcomes [32]. The ability of nanoparticles to encapsulate the drug is primarily determined by their high surface-to-volume ratio, amphiphilic character, porous or core–shell structure, and surface functionalization. These properties enable efficient drug–carrier interactions, high loading capacity, and improved physicochemical stability, while surface modification further enhances encapsulation efficiency and controlled release behavior [37,38].

**Figure 2:**
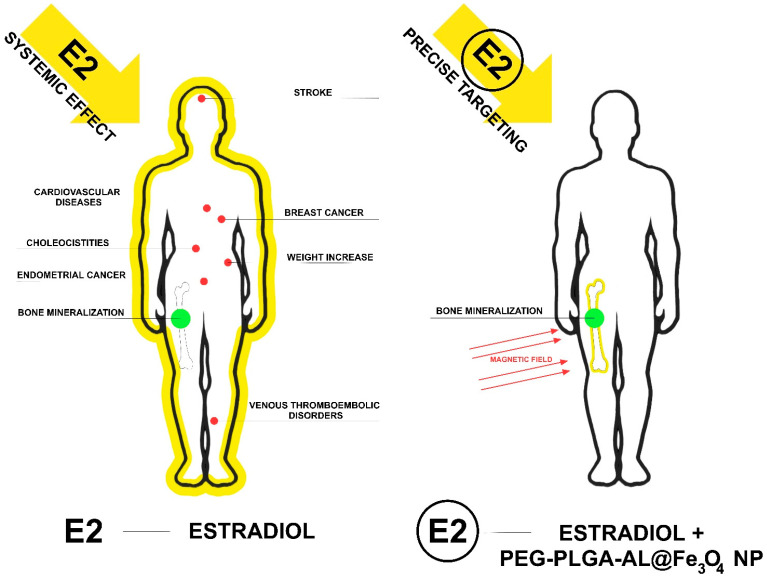
Hypothesis regarding the expected effect of estradiol in combination with nanoparticles—PEG-PLGA-AL@Fe_3_O_4_ NP. The figure was prepared based on [18].

Figure 2 shows the authors’ hypothesis regarding the expected effects of estradiol and estradiol combined with PEG-PLGA-AL@Fe_3_O_4_ NP+MF nanoparticles [18]. This hypothesis concerns the reduction in side effects associated with the use of E2, but it has not yet been confirmed by research.

### 3.3. Nanoparticles Used in E2 and RLX Therapy in Recent Years

#### 3.3.1. Lipid Nanoparticles (LNPs)

They include lipid-based structures, including liposomes, solid lipid nanoparticles (SLNs), nanostructured lipid carriers (NLCs), nanoemulsions, and other nanostructured lipid forms, including nanogels. All of these systems utilize lipids as the main carrier component, but differ in lipid phase composition, physical state, and pharmacokinetic properties [39,40,41]. NLC nanoparticles are a second-generation drug delivery method in which the solid lipid is partially replaced by liquid lipids [42]. Nanoemulsions are oil-in-water lipid systems in which lipophilic active substances, e.g., RLX and levomeloxifene (L-ORM), are dissolved in an oil phase dispersed in water, which significantly increases their bioavailability after oral administration [40].

##### RLX-NLC

RLX was encapsulated in an amorphous form within the lipid core, which allowed for a high degree of encapsulation. Nanostructured lipid carriers (NLCs) supplemented with RLX were administered orally to female Wistar rats to enhance oral bioavailability and improve stability. Rats were divided into two groups: the control (RLX suspension) and the test RLX-NLC groups. The administration volume was 15 mL/kg, and the doses were 15 mg/kg in all control group animals. Blood samples for analysis were collected by puncture of the orbital venous plexus. Cells were analyzed for cumulative drug release to measure the amount of RLX released under gastrointestinal pH conditions. By using NLC, minimal RLX release was achieved under gastrointestinal pH conditions. Studies have shown that NLCs have the greatest stability at 4 °C [25]. Additionally, it was described that the crystallinity of RLX was significantly reduced due to the dispersion of raloxifene in the lipid–oil matrix compared to the control group. RLX-NLC also showed improved oral bioavailability compared with the RLX suspension. The authors attribute this to low drug release in the gastrointestinal tract. Therefore, the use of lipid nanoparticles could also improve the pharmacokinetic parameters of RLX [25].

##### RLX-NLCs

In this study, a lipid core composed of monostearate + vitamin E and RLX was used. The phospholipid shell was additionally coated with a surfactant. The drug was administered orally to female Wistar rats. The study animals were divided into three groups, each receiving I—0.25% carboxymethylcellulose solution; II—RLX-NLC suspension; and III—suspension of the marketed RLX tablet, i.e., Ralista^®^. Doses of RLX were calculated for each rat based on the human oral administration. Doses were taken at pre-defined time intervals of 0.083, 0.25, 0.50, 1, 2, 4, 8, 12, 24, 48, and 72 h from each rat via the orbital plexus. The pharmacokinetic profile was assessed and showed that the drug release profiles for NLCs indicate a prolonged release of RLX, apparently due to the high melting point of the solid lipid, which ultimately leads to slower dissolution of the matrix. The analysis demonstrated improved pharmacokinetic parameters, including increased time to peak concentration (Tmax), maximum plasma concentration (Cmax), and magnitudes of model-independent parameters such as area under the curve (AUC_0–72 h_), K, Ka, T1/2, and mean residence time (MRT), for RLX-NLC compared to RLX alone [43]. These changes in pharmacokinetic parameters suggest that the lipid matrix may enhance the biopharmaceutical properties of RLX compared with commercially available tablets. This may have an impact on the hepatic metabolism of the developed RLX-NLC cells. In vitro studies demonstrated that the cytotoxicity and proliferation of osteoblastic MG-63 cells improved with RLX-NLC compared to the other groups. Furthermore, it was demonstrated that RLX-NLCs were readily internalized in the tested cells. It has also been described that optimal long-term storage of RLX-NLC must be under refrigerated conditions (i.e., 5 ± 3 °C) [43].

##### L-ORM and RAL

An oil-in-water nanoemulsion was developed as a carrier for L-ORM and RAL. The drugs were dissolved in the oil phase and stabilized using surfactants and co-surfactants, allowing for the formation of particles in the nanometer range. The studies used MC3T3-E cells and female SD rats, which were orally administered a nanoemulsion at a dose of 5 mg/kg equivalent of L-ORM and 10 mg/kg equivalent of RAL, relative to the animal body weight, to determine the pharmacokinetic profile. After oral dosing of free drug solutions and nanoformulation, blood samples (200–250 μL per rat) were collected from the retro-orbital plexus for each time point: 0.08, 0.25, 0.5, 1, 2, 6, 12, 24, 48, 72, 96, and 120 h. The nanoformulation increased the oral bioavailability of both drugs (L-ORM and RAL), and improvements were observed in several pharmacokinetic parameters, including Cl, Cmax, AUC_0–last_, AUC_0–∞_, Vd, T1/2, and Tmax. A stable nanoformulation with the simultaneous loading of two drugs—L-ORM and RAL—in the oil core of the nanocarrier was developed and the analytical method used (LC–MS/MS) was confirmed as suitable for the assessment of the pharmacokinetics and bioavailability of both substances [40].

##### RLX-Spanlastic Nanogel

The study used a transdermal nanogel based on Carbopol 934, in which spanlastic nanocarriers (spanlastic vesicles) containing RLX were dispersed. Carbopol 934 was dispersed in double-distilled water, and then RLX vesicles and triethanolamine were added to the mixture, which acted as a neutralizer and enabled the formation of a transparent, viscous nanogel with properties suitable for transdermal administration. The resulting nanogel was applied transdermally to Wistar rats. The RLX dose in the nanogel was 31 µg. The animals were divided into three groups: I (control), II (positive control) treated with an aqueous solution of formalin (0.8% *v*/*v*), and III (animals treated topically with the optimized RLX-spanlastic nanogel). Freshly prepared formalin solution and nanogel were applied to the backs of rats from each group and monitored at various time points (1 h, 24 h, 72 h, and the seventh day) for erythema and edema scores. Tail veins were also collected to determine the pharmacokinetic profile. The results indicated improved oral bioavailability without significant changes in biochemical parameters after transdermal administration of RLX-spanlastic nanogel, and changes in pharmacokinetic parameters, including increases in Tmax, Cmax, magnitudes of model-independent parameters such as area under the curve (AUC_0–48_), AUC_0–∞_, and T1/2 compared with the aqueous solution [41]. The preparation also showed no signs of skin irritation and did not cause any significant changes in skin histology. To assess cytotoxicity, rats treated with normal saline, RLX-oral suspension (1 mg/kg), and RLX-spanlastic gel (1 mg/kg) once daily, separately, were compared. Animals were monitored for general appearance, toxicological signs, and behavioral and clinical abnormalities twice daily for 14 days. Additionally, animals were euthanized; a portion of skin tissue was collected, as well as other vital organs, including the heart, kidneys, and skin. Acute toxicity assessment of the drug, as well as the formulation itself, revealed no toxic effects or hematological changes in treated rats. The results showed that the nanogel has good occlusive properties in vitro as well as a good texture profile value. Ovariectomized rats were used to determine parameters associated with osteoporosis. The results showed that, including bone volume (BV/TV), trabecular separation (Tb.Sp), trabecular number (Tb.N), and connectivity density (Conn.Dn), transdermal RLX delivered via spanlastic nanogel resulted in improved parameters compared with RLX suspension. Increased bone mineral density (BMD) measured by quantitative computed tomography (QCT) and increased creatinine (Cr) levels were also demonstrated [41].

#### 3.3.2. Protein Nanoparticles

They are created by assembling several native or modified proteins into nanosized assemblies. Although proteinaceous materials constitute only a small percentage, their interesting properties, such as high biocompatibility, complete degradability, and specific interactions with receptors, make them an interesting material for the development of the next generation of nanoparticle drug delivery systems [44]. Human serum albumin (HSA) is one of the most abundant endogenous proteins in human blood. It can be used as a natural drug carrier because it binds to many metabolic compounds and therapeutic drugs. It has been used as a drug carrier in injectable formulations with great success and versatility. It increases the compatibility and half-life (T1/2) of nanocarriers in the blood [24,27].

##### RAL/VitaD/HSA and RAL/VitaD/HSA/PSS NPs

The study utilized HSA-based nanoparticles encapsulating RAL and vitamin D_3_. The carrier was stabilized with sodium polystyrene sulfonate (PSS). Raloxifene and vitamin D3 nanoparticles (RAL/VitaD/HSA and RAL/VitaD/HSA/PSS nanoparticles) were developed by lyophilization and hydration. The resulting nanoparticles are stable at 4 °C for 4 weeks. A hemolysis test demonstrated that even after increasing the raloxifene treatment concentration to five times the therapeutic dose (100 µg/mL), the NPs used demonstrated satisfactory blood compatibility. In the study, nanoparticles were administered intravenously to female Wistar breeds. The rats were injected with the RAL/VitaD/HSA/PSS NPs (5 mg of raloxifene/kg of body weight) via the tail vein. Concomitantly, the triturated EVISTA (raloxifene 60 mg film-coated tablets) was suspended in PBS and then fed to rats via oral gavage at a dose of 30 mg of raloxifene/kg of body weight, as a control group. A blood sample was then taken from the tail vein. The applied nanoparticles have been reported to significantly improve the pharmacokinetics of raloxifene by increasing Cmax, area under the plasma concentration versus time curve (AUC_0–t_), and MRT. A decrease in Tmax and clearance parameters (CL/F) and a significantly lower volume of distribution parameter (Vd/F) were also reported compared to a specially developed intravenous solution. Additionally, a human foreskin fibroblast cell line (Hs-68, ATCC: CRL-1635™) and a human osteosarcoma cell line (MG-63, ATCC: CRL1427™) were used. It was also shown that the designed nanocarriers can reduce the direct toxicity of loaded drugs on cells, and the simultaneous loading of raloxifene and vitamin D3 in Ral/VitaD/HSA/PSS nanoparticles reduces raloxifene toxicity in cells by controlling its release from the nanoparticles [24]. This means higher bioavailability and longer duration of action, thus reducing the risk of side effects and making it a potentially more effective alternative to oral therapy. It was also shown that the application of RAL/VitaD/HSA/PSS NP in MG-63 cells showed significant induction of alkaline phosphatase (ALP) activity and the cells showed a time-dependent increase [24].

##### RAL/HSA/PSS NP

RAL was encapsulated in prepared RAL/HSA/PSS NPs to develop an intravenous formulation that bypasses intestinal glucuronidation and first-pass metabolism, which may improve bioavailability. Studies also examined stability and found no significant differences in particle size of the RAL/HSA/PSS nanoparticles, even after re-lyophilization and storage at room temperature for 28 days. This is important because the pharmaceutical formulation must have good storage stability and shelf life. The preparation was injected intravenously into Wistar rats using a lyophilization-hydration process. The rats were divided into two groups: one group received an EVISTA suspension (30 mg of RAL/kg) by oral gavage, and the other group received the RAL/HSA/PSS NPs (5 mg of RAL/kg) via IV injection. Administration of RAL/HSA/PSS NPs resulted in higher plasma exposure and slower release of raloxifene, leading to a prolonged half-life and allowing for lower dosing [27]. Studies have shown that oral administration of EVISTA tablets allowed detection of RAL in plasma only at a dose of 30 mg/kg. However, after intravenous administration, a dose of 5 mg/kg of RAL in the RAL/HSA/PSS NP formulation was sufficient to achieve measurable plasma concentrations. Furthermore, the Cmax of raloxifene in the RAL/HSA/PSS NP group was 10.8 times higher than in the EVISTA group at 4 h post dose, even though the raloxifene dose in the RAL/HSA/PSS NP group was significantly lower compared to the EVISTA group. Moreover, the Vd/F of NP RAL/HSA/PSS was significantly lower than that of EVISTA, indicating that intravenous administration of NP RAL/HSA/PSS may prevent the rapid spread of raloxifene into tissues, thereby reducing the risk of adverse events. For the hemocompatibility test, heparin-stabilized blood samples were collected from Wistar rats. To each 200 μL blood sample, 200 μL of RAL/HSA/PSS nanoparticles were added. RAL concentrations were 10, 25, 50, 75, 100, 200, 300, 400, and 500 μg/mL. Studies were also conducted in vitro on the Human newborn normal diploid fibroblast cell line (Hs-68) and human osteosarcoma cell line (MG-63). Cells were incubated in fresh medium supplemented with RaI/HSA/PSS NPs and free raloxifene at concentrations of 0, 1, 10, 25, and 50 μg/mL for 24 and 72 h. The viability of Hs-68 cells was calculated compared to the control group. The studies showed that free RAL exhibited potent cytotoxicity at concentrations above 25 μg/mL. However, the viability of cells treated with RaI/HSA/PSS nanoparticles always exceeded 90%, even at higher RAL concentrations (50 μg/mL), indicating that the designed nanoparticles can reduce the toxicity of high raloxifene doses in both Hs-68 and MG-63 cells [27]. Additionally, the tested nanoparticles did not cause hemolysis, even at a very high concentration (100 µg/mL), which is five times higher than the therapeutic dose. The results indicate reduced distribution into peripheral tissues compared with EVISTA. Intravenous administration of RAL/HSA/PSS nanoparticles bypasses intestinal glucuronidation and first-pass metabolism, which may increase bioavailability, prolong half-life, and allow for lower dosing in the treatment of postmenopausal osteoporosis. Bioavailability was >2% [27].

#### 3.3.3. Polymer Nanoparticles (PNPs)

They are nanoparticles derived from polymer building blocks. Their advantage lies in the ability to control size, shape, and surface charge, which is advantageous for drug delivery. Equally beneficial, however, is the vast variety of chemical and biological functions that can be incorporated into polymer nanoparticle designs. Such advanced functionality has led to the development of nanoparticles with functionalities that may respond to specific biological environments and enable controlled drug release [45]. They are divided into nanospheres and nanocapsules. Nanospheres are structures in which the drug is homogeneously dispersed within a polymer matrix, forming a compact, solid particle. Nanocapsules, on the other hand, have a core–shell structure, where the active substance is encapsulated and surrounded by a thin polymer layer. This classification is based on the internal architecture of the particles and the mechanism of active substance release [23].

##### RLX-PNPs

The study used RLX encapsulated in a chitosan-TPP matrix obtained by ionic gelation. Chitosan served as the cationic polymer carrier, while pentasodium triphosphate (TPP) was the anionic cross-linking agent. The preparation was administered orally to SD rats via gastric tube (30 min, 50 min, 1 h, 2, 3, 4, 6, 12, and 24 h). The rats were divided into two groups. One group received an RLX suspension, and the other group received RLX-PNP at a dose of 5 mg/kg.

Cell viability when exposed to the described nanocarriers was above 80% [23].

In the second part of the experiment, the rats underwent ovariectomy and were divided into three groups: I—oral administration of 1.4 mg/kg RLX-PNP; II—oral administration of 5.6 mg/kg RLX suspension; and III—no preparation was administered, serving as an untreated control. Treatment continued for 28 days. Blood was withdrawn from the cannulated femoral artery. The results demonstrated the crystalline nature of pure RLX, while its incorporation into RLX-PNPs indicated conversion of the crystalline drug to an amorphous form. A significantly controlled release of RLX from PNPs was observed due to the polymer effect. Furthermore, the nanoparticles were shown to be stable at 4 °C for 6 months. The use of RLX-PNPs in PNPs significantly improved drug bioavailability through improved solubility and reduced serum biochemical markers of ALP and calcium (Ca). Improved pharmacokinetics were also reported, with increased Tmax, Cmax, and (AUC_0–∞_) compared to pure RLX treatment. Furthermore, RLX-PNPs were associated with markers indicating reduced bone resorption and enhanced bone formation [23]. This study showed that in the case of RLX-PNP application, the level of fibrils bound to pyridinoline (Pyr) and deoxypyridinoline (DPyr) is significantly reduced, which play an important role in the covalent binding of collagen molecules in the bone matrix and influence bone metabolism [23].

#### 3.3.4. Inorganic Nanoparticles (iNPs)

They consist of inorganic atoms linked by covalent or metallic bonds. The main core of iNPs is formed by the crystallization of inorganic salts, arranged three-dimensionally by bonded atoms. As a result, the particles are highly organized and ordered, which increases their resistance to destabilizing factors [30]. Hydroxyapatite (HA) (Ca_10_(PO_4_)_6_(OH)_2_) is the main chemical component of skeletal bones and is converted to Ca^2+^ and PO_4_^3−^ in the body. It supports osteoblast activity and is involved in bone matrix formation. Probably by increasing osteoblast adhesion and proliferation, it supports bone development and osseointegration and is therefore widely used in orthopedics. The main advantages of hydroxyapatite are its biocompatibility and high in situ biodegradability. Methods for obtaining hydroxyapatite nanoparticles with admixtures of other elements, e.g., ZnHA-NPs, are also being developed to achieve even better therapeutic effects [19]. Although conventional hydroxyapatite is nonporous, Zn-doped hydroxyapatite nanoparticles can encapsulate drug molecules by adsorption on the surface and in the interlayer spaces. As demonstrated for HAp-methotrexate complexes, the interlayer structure and increased adsorption surface area enable higher loading capacity and prolonged drug release, with the nanoparticle morphology influencing loading efficiency and release kinetics. This mechanism highlights the potential of ZnHA nanoparticles as effective drug carriers, enabling controlled delivery of therapeutic compounds such as estradiol (E2) or raloxifene [46].

Application in preclinical studies:

Nano Zinc Hydroxyapatite (ZnHA-NPs) + E2

Ovariectomized female SP rats were used in the study. The animals were divided into seven groups: 1—control group (SHAM-operated); 2—ovariectomized (OVX) group; 3—OVX plus estradiol therapy (20 μg/kg (OVX/E2)) group; 4—OVX plus ZnHA-NP therapy (500 μg/kg (OVX/ZnHA)); and 5—a combination treatment of E2 (10 μg/kg plus ZnHA-NPs (250 μg/kg) (OVX/E2 + ZnHA)) group. E2 was administered orally while ZnHA was injected intravenously twice during the experiment. The simultaneous administration of E2 and ZnHA nanoparticles acts synergistically, which was supposed to contribute to the increase in the therapy efficacy [19]. At the end of the experiment, blood samples were collected from the medial canthus of the eye of each rat from the groups, and histological analysis and gene expression were also performed as well as bone density measurement. The experiment showed that the administration of ZnHA-NPs in monotherapy or with E2 was more effective than the treatment with E2 only in terms of bone turnover markers (Ca, Phosphorus (P), ALP, and N-terminal propeptide of type-I procollagen (PINP) levels). Furthermore, E2 and/or ZnHA therapy resulted in a decrease in RANKL expression and an increase in OPG expression in the femur. However, treatment with both OVX/E2 and OVX/E2 + ZnHA resulted in a lower mRNA level of femoral estrogen receptors (ER-α and ER-β) with an increased expression of osteocalcin (OC) compared to OVX rats, which indicates increased bone mineralization. It was also reported that the combined treatment with E2 and ZnHA-NPs was more effective in reducing cytokine levels than the use of each of them separately at higher doses. This may indicate the possibility of long-term use of estrogen at low doses with ZnHA-NPs in the prevention or treatment of osteoporosis. NO levels were significantly lower in rats from the OVX + E2 + ZnHA group than in the OVX group. Restoration of histological changes in cancellous and cortical bone, an increase in the concentration of vascular endothelial growth factor (VEGF) and proliferating cell nuclear antigen (PCNA), and an increase in collagen density were also described [19].

#### 3.3.5. Exosomes

These are natural nanoparticles that participate in intercellular communication through molecular transport. They possess a membrane-like vesicular structure and a proteinaceous surface, making them widely used in drug delivery research. Exosomes combine the advantages of both types of pharmacotherapy systems, i.e., synthetic drug carriers and cellular drug delivery systems, while simultaneously avoiding the disadvantages of both. Compared to synthetic carriers, exosomes prevent rapid drug clearance from the body, are characterized by high biocompatibility, exhibit no toxic effects, and do not trigger an immune response.

Application in preclinical studies:

Exosomes

The study was conducted on three male New Zealand white rabbits, from which bone marrow was harvested from the femur and tibia. The cells were then cultured, and mesenchymal stem cells (BMMSCs) in the third passage were used in the experiments.

E2-loaded exosomes were placed in these cells.

A significantly increased survival rate was demonstrated in BMMSCs treated with E2-loaded exosomes compared to the control group and E2-only cells (almost 10% higher survival rate). The authors suggest that exosomes tend to act and move to inflammatory areas, such as bone tissue, resulting in very precise drug targeting. However, this study represents only basic and preliminary research in the field of nano-sized 17β-estradiol drug-targeting systems, which could potentially be used as a precise drug delivery method for the treatment of various diseases, including osteoporosis [20].

#### 3.3.6. Hybrid Nanoparticles

They are a class of advanced drug carriers that combine different types of materials in a single structure. An example used in recent years for drug delivery is hybrid polymer-lipid nanoparticles (RLX-bNPs), composed of a functional polymer and a biocompatible lipid. These nanodrug carriers combine the beneficial properties of both polymer and lipid nanocarriers. Unlike single polymer nanoparticles, the lipid component facilitates stable drug encapsulation, while the polymer provides the nanoparticles with functionalities such as enhanced stability, accessory bioadhesion, and controlled release [26]. Another example of hybrid nanostructures are inorganic-polysaccharide microspheres based on hydroxyapatite substituted with magnesium, silicon, and cobalt (Mg, Si, Co-HA) and combined with sodium alginate. The structure of this system combines the properties of bioactive hydroxyapatite, which supports bone regeneration, with the hydrogel-like, biodegradable nature of alginate, which enables controlled drug release and its local anabolic effect in the place of bone defect [47]. Other nanoparticles that may aid in the treatment of postmenopausal osteoporosis are superparamagnetic Fe_3_O_4_ nanoparticles. The multifunctional water-soluble polymer PTMP-PMAA was used as the ligand. The resulting PTMP-MAA@Fe_3_O_4_NP suspension is characterized by high magnetization, stability, and low cytotoxicity. The use of polyethylene glycol (PEG) ensures improved stability of the nanoparticles in the bloodstream. These studies also utilized a magnetic stimulation technique by applying a rotating magnetic field source to the brain. Fe_3_O_4_ nanoparticles have unique supermagnetic properties and can therefore also be used in targeted therapies [3]. Supermagnetic Fe_3_O_4_ nanoparticles were also used in combination with PGLA modified with alendronate (AL) via chemical bonds to target bone. This modified PGLA was used as a matrix in which sex steroid hormones (E2) and magnetic Fe_3_O_4_ NPs were incorporated. Both PGLA and Fe_3_O_4_ NPs have been approved for clinical use by the FDA due to their good biocompatibility. This system allows for drug delivery by magnetically controlling drug release under the control of an external magnetic field. The bioavailability of these nanoparticles was tested to be >2% [18].

Application in preclinical studies:

Polymer/Lipid Hybrid Nanoparticles—RLX-bNP Lipid Nanoparticles—RLX-cNP

Hybrid polymer-lipid nanoparticles were used in the study. SD rats were divided into three groups. Each group was administered RLX suspension at a dose of 25 mg/kg by gavage, in the form of I—dispersed in 0.5% CMC–Na solution (RLX·HCl); II—common lipid nanoparticles (RLX-cNPs); and III—bioadhesive nanoparticles (RLX-bNPs). At the predetermined intervals (0.5, 1, 2, 4, 6, 8, 12, 16, and 24 h), tail vein blood samples were analyzed, which enabled the assessment of pharmacological profiles. When formulated as lipid nanoparticles, intestinal absorption of RLX was significantly improved, with Cmax, Tmax, and area under the plasma concentration versus time curve (AUC_0–t_) significantly increased compared to RLX·HCl. It was also demonstrated that the nanoparticles resulted in higher drug concentrations in blood and improved absorption and bioavailability of RLX from NPs compared to RLX·HCl [26]. Furthermore, fluorescent staining demonstrated the presence of RLX-bNP throughout the intestine, indicating good epithelial permeability or affinity. The good bioadhesion of RLX-bNPs may increase the chance of contact and prolong the retention time of the charge on the absorptive epithelium, which is beneficial for subsequent penetration. This is also the mechanism underlying the excellent intestinal absorption of RLX-bNPs, which translates into increased bioavailability. The release of RLX from nanoparticles, which depends on the environment, was also assessed. RLX-bNP was the lowest of the three forms tested in the 0.1 M HCl environment. Furthermore, in the three different media, the cumulative release of RLX-bNP did not exceed 42% within 8 h, which is similar to gastrointestinal transport. Sustained release allows for the entrapment of most RLX molecules within the nanoparticles and transport through the gastrointestinal tract, which is beneficial for RLX absorption via intact nanoparticles. Therefore, the use of nanoparticles increased the bioavailability of RLX [26]. Furthermore, strong intestinal adhesion and permeability were demonstrated. The experiment was repeated on Caco-2 cells to evaluate the cellular uptake of 50 μg/mL free RLX (solution), RLX-cNP, and RLX-bNP. Cells were incubated with the drugs for 0.5, 1, and 2 h at 37 °C, respectively. Cellular uptake of RLX significantly increased when loaded in both RLX-cNP and RLX-bNP nanoparticles compared to the free form. Both of these lipid carriers also had high affinity for enterocytes. RLX-bNP also exhibited good bioadhesion [26].

PEG-PLGA-AL@Fe_3_O_4_/E2 NPs + MF

Alendronate-modified PLGA (AL) was used in the NP to target bone. After E2 and Fe_3_O_4_ were incorporated into PGLA, it was administered intravenously into the tail of female Sprague-Dawley (SD) rats once a week 9 times. The nanoparticles were administered in various combinations (TG1-OVX, TG2-PEG-PLGA-AL@E2 NPs, TG3-PEG-PLGA-AL@Fe_3_O_4_ NPs, TG4-PEG-PLGA-AL@Fe_3_O_4_/E2 NPs). E2 in each form was administered at an E2 dose of 63 μg/kg. In this study, an electromagnetic field (MF) was additionally used in TG5 to generate heat, which enhances E2 release in bone. It was found that NPs labeled with 1,1-dioctadecyl-3,3,3,3-tetramethylindotricarbocyanine iodide (DiR iodide) in PEG-PLGA-AL@Fe_3_O_4_/DiR accumulated mainly in bone. Then, based on three-dimensional reconstruction, bone volume BV/TV, trabecular thickness (Tb.Th), trabecular number (Tb.N), trabecular spacing (Tb.Sp), and bone markers (PINP, osteocalcin (OC), C-telopeptide (CTX), TRAP-5b) were calculated. Body and uterine weights were also measured, and sections of the main organs (liver, uterus, and small intestine) were examined. Based on the results, it was concluded that treatment with PEG-PLGA-AL@Fe_3_O_4_/E2+MF more effectively improved bone volume and structure by increasing BV/TV, PINP, and (OC) and decreasing Tb.N, Tb.Sp, C-telopeptide (CTX), and 5b-tartrate-resistant acid phosphatase 5b (TRAP-5b) compared to free estrogen and nanoparticle therapy [18]. Furthermore, administration of PEG-PLGA-AL@Fe_3_O_4_/E2 nanoparticles resulted in a lower increase in uterine weight in ovariectomized rats (OVX) compared to the group treated with free E2. E2 also improved bone strength. Organ analyses demonstrated the lack of toxicity of the nanoparticles used. This may be beneficial in minimizing the side effects of estrogen therapy. In the next step, the studies were carried out on the murine macrophage cell line (Raw 264.7) and human umbilical vein endothelial cells (HUVECs), thanks to which it is known that PEG-PLGA-AL@Fe_3_O_4_/C6 NP particles are effectively absorbed by Raw 264.7 cells. Additionally, the use of MF resulted in an increase in the release and bioactivity of E2 intracellularly. The cytotoxicity of the tested cells was described as above 90%. The stability and biocompatibility of the engineered NPs were described during the study. In vitro drug release, cellular uptake capacity, and targeting effects were also investigated [18].

PTMP-MAA@Fe_3_O_4_ NP

Four groups of SP rats were used in the study. I—YC (young control). II—postmenopausal rats (MC). III—estrogen treatment (ET) group. Animals were treated intragastrically with 0.2 mL/100 g of body weight, once daily, for 4 weeks. IV—osteoporosis prevention (OP), prepared PTMP-MAA@Fe_3_O_4_ NPs, diluted to 2.45 mg/mL, were administered in a 200 nL amount by microinjection into the paraventricular nucleus of the hypothalamus of rats. After administration of the suspension with supermagnetic nanoparticles, a rotating magnetic field (50 mT, 2 × 1 h daily for 8 weeks) was applied to the brain to magnetically stimulate the nerves. Magnetic stimulation is a noninvasive method that can directly stimulate the cerebral cortex and has a low impact on intermediate tissues. Increases in serum estrogen concentrations, improved bone mineral density, bone mass, and bone markers were observed. Wet and dry humeral bone mass in Group II and Group IV increased significantly compared to the other study groups. This method also regulates bone metabolism in perimenopausal rats, balances bone formation and resorption in rats, and subsequently alleviates osteoporosis. In this study, it was found that the level of oxytocin (OT) increased in the ET and OP groups, which may directly regulate bone metabolism. In the serum of rats from the ET and OP groups, the serum levels of PTMP-MAA@Fe_3_O_4_ NPs increased, indicating that neuromagnetic stimulation based on superparamagnetic NPs can promote bone differentiation and formation in perimenopausal rats. The serum levels of Boneglaprotein (BGP), type I collagen carboxy-terminated cross-linked peptide (CTX-I), and TRACP-5b were significantly decreased in the ET and OP groups. Furthermore, the biocompatibility and low cytotoxicity of PTMP-MAA@Fe_3_O_4_ NPs were also demonstrated. No significant pathological changes were observed in brain tissue samples from either group. Therefore, the injection of PTMP-MAA@Fe_3_O_4_NP suspension into rats was considered safe [3].

CS/KER-RAL, CS-RAL

Structures composed of hydroxyapatite nanocrystals modified with magnesium and silicon ions (nanocrystalline, magnesium, and silicon co-substituted hydroxyapatite) (Mg, Si-HA) were used. The composite microspheres were suspended in an organic matrix prepared from a sodium alginate salt solution with the addition of chondroitin sulfate (CS) or keratin (KER). During microsphere formation, RAL hydrochloride (RAL HCl) was introduced into the matrix and uniformly dispersed within the structure. The entire structure was then cross-linked with Mg^2+^ ions, creating stable, porous composite microspheres. The resulting granules are materials with favorable parameters, enabling gradual, local delivery of therapeutic substances to the immediate surroundings of the diseased tissue. CS/KER-RAL beads, consisting of both CS and KER, were characterized by the most prolonged drug release and no burst release effect compared to single-component samples (CS and KER). In vitro studies were conducted on human osteosarcoma (Saos-2) and human fetal osteoblast (hFOB 1.19) cells. The cytotoxicity of 48 h exposure to Cs-c, CS/KER, CS-RAL, and CS/KER-RAL granules was assessed and showed that the released RAL from both CS-RAL and CS/KER-RAL granules was not toxic to human osteoblasts but caused a significant decrease in tumor cell viability [47]. Table 2 presents the nanoparticles used in preclinical studies on E2 and RLX delivery for the treatment of postmenopausal osteoporosis in the last 5 years (2020–2025).

## 4. Comparison of Nanoparticles Described in the Context of Osteoporosis Treatment in 2020–2025

Each of the currently used nanocarrier systems has significant advantages and disadvantages that determine its therapeutic efficacy and safety. Lipid nanoparticles have attracted particular attention in recent years, as they are the most promising drug delivery systems [48]. Among nanoparticles, liposomes are currently the only type approved by the FDA for clinical use [49].

The undoubted advantages of these nanoparticles include their high capacity for encapsulating hydrophilic or lipophilic drugs, high biocompatibility, controllability, targeted release of the active substance, and many others [48]. NLC nanoparticles are a second-generation drug delivery method in which the solid lipid is partially replaced by liquid lipids. As a result, NLCs provide increased storage stability and drug loading capacity due to impaired crystallite formation [42]. However, there are concerns that the inclusion of additional materials in the development of NLC formulations may lead to higher product prices. Furthermore, they may cause skin irritation, and converting the liquid form to a solid is difficult to achieve. This is undoubtedly a disadvantage, as the solid form is essential to maximizing the potential of NLCs as a drug delivery system [50].

Another example of nanoparticles proposed in combination with osteoporosis drugs is polymer nanoparticles (PNPs). The advantages of PNPs also include their ability to control drug release and protect contained drugs from the microenvironment of diseases and interactions of complex biological systems of the human body, and may improve drug stability, bioavailability, and therapeutic performance [23]. Poly(lactic-glycolic acid) (PLGA) is a versatile synthetic polymer widely used in the pharmaceutical sector due to its biocompatibility and biodegradability [51].

A potential drawback is that, despite their biocompatibility, some polymer degradation products, e.g., PGLA, may be harmful in higher doses or accumulate in specific organs. Difficulty in improving drug release and encapsulation is also a significant obstacle [48].

Another type of nanoparticle presented in the described articles is protein nanoparticles, particularly human serum albumin (HSA). It has been used as a drug carrier in injectable formulations with great success and versatility. It increases the compatibility and half-life (T1/2) of nanocarriers in the blood [24,27]. However, there are concerns that protein nanoparticles are characterized by low drug loading efficiency [52].

Inorganic nanoparticles are considered easy to synthesize. Surface functionalization and good stability are also possible to obtain. However, according to the literature, their disadvantage is the nonbiodegradable toxicity coating required [52].

Exosomes compared to cellular drug delivery systems demonstrate more precise drug transport and targeting, and are free of clinical complications [20]. They possess all the potential and characteristics expected of an effective drug carrier. These include adequate drug loading capacity, high biocompatibility, the ability to release the loaded drug, easy surface receptor modification, and the ability to cross tissue and physiological barriers within the body at their nanoparticle size [20].

Hybrid nanoparticles combine the advantages of different materials, such as stable drug encapsulation from lipids and enhanced functionality, controlled release, and bioadhesion from polymers. However, their complex structure may increase production costs and pose challenges for large-scale standardization and regulatory approval.

Among the analyzed papers, only the article on PLGA-AL@Fe_3_O_4_/E2 NPs + MF described a targeted effect on bone in vivo [18]. The authors of the analyzed papers hypothesize that the use of nanoparticles with drug delivery may ensure targeted delivery of active substances to specific tissues [3,18,26].

Most studies describe the encapsulation (EE) of the obtained formulations. These studies indicate that the use of hybrid nanocarriers allows for the highest efficiency. For example, (E2) and Fe_3_O_4_ magnetic NPs were encapsulated into PLGA-based matrices, resulting in relatively good encapsulation (58%) [18]. Additionally, in some of the presented studies, the measured EE value exceeded 90%, which is undoubtedly a very good result [23,25,26]. Among the available articles from recent years, only the PLGA-AL@Fe_3_O_4_/E2 NPs + MF and RAL/HSA/PSS NPs have been tested for bioavailability [18,27]. Their bioavailability was >2%, which is still a relatively unsatisfactory result. Toxicity has only been verified in studies [18,23,27], which significantly limits the ability to draw robust and generalizable conclusions regarding the safety of the remaining nanocarrier systems. Systematic evaluation of cytotoxicity and hemocompatibility is essential to enable meaningful comparison between platforms and to support translational relevance. Table 3 presents the most important advantages of the nanoparticles discussed in this article.

Among the analyzed nanocarrier platforms, liposomes appear to be the best candidates for clinical studies, as they are the only FDA-approved nanoparticles and exhibit high biocompatibility and translational feasibility. Additionally, in recent years, research has shown great potential of lipid nanoparticles in combination with mRNA therapy [53], which gives great hope for their use in the treatment of osteoporosis. In contrast, other nanocarriers, including hybrid and biologically derived systems, although highly promising in the preclinical stage, currently lack sufficient safety and bioavailability data to support clinical implementation.

## 5. Challenges in the Development

The authors of the presented studies suggest that thanks to the use of HA, it is possible to precisely direct biomolecules to the target tissue [36]. However, these hypotheses are not supported by scientific research. It has been shown that HA-based nanocarriers can increase the preferential accumulation of biomolecules in bone tissue due to their affinity for the mineralized matrix. However, truly precise or tissue-specific delivery has not yet been conclusively demonstrated in vivo. Therefore, recent publications do not support the claim of precise targeting [49]. Furthermore, a review [54] discusses nanoparticle strategies for bone, taking into account affinity for hydroxyapatite and bone and improved bone accumulation. However, the authors do not claim precise or exclusive bone targeting. The positive effect of the nanoparticles used on improving bone parameters may only result from improved pharmacokinetics of the drugs used. This is indicated by the fact that all studies describing the combination of RLX with nanoparticles showed improved pharmacology compared to control groups in which nanoparticles were not used [23,25,26,27,41,43]. Future studies should therefore include more rigorous biodistribution analyses, quantitative tissue-level comparisons, and standardized targeting efficiency metrics to clearly distinguish between preferential accumulation and true targeting. At this point, interpretation of reports of precise bone delivery should be undertaken with caution, as the lack of such data limits the ability to fully assess the translational potential of nanocarrier-based E2 and RLX therapies.

A significant limitation of the analyzed studies is the lack of systematic data on the long-term toxicity and potential immunogenicity of the nanoparticles used. In most studies, safety assessment is limited to short-term in vitro tests or observations of acute tolerability, which does not allow for a comprehensive assessment of the risk profile in the context of chronic osteoporosis therapies. Furthermore, available biodistribution data are fragmented and often based on indirect markers, making it difficult to draw clear conclusions about the fate of nanocarriers in vivo. Additional challenges include the heterogeneity of the animal models used, the variety of routes of administration, and the lack of standardization of experimental parameters, which limits the comparability of results between studies. It should also be emphasized that most of the available data comes from preclinical studies, and their extrapolation to clinical settings remains limited. Considering these aspects in future studies will be crucial for a reliable assessment of the translational potential of E2- and RLX-based nanocarrier therapeutic strategies.

Nanoparticle-based formulations may enhance the biological activity of E2 and RLX not only by improving their pharmacokinetic profiles but also by influencing cellular uptake and local availability of the hormones at the bone surface. Their physicochemical properties may modulate key pathways involved in bone remodeling, including RANKL/OPG signaling, ALP activity, and the oxidative stress response. However, current evidence suggests that these effects are primarily due to prolonged systemic exposure rather than to actual bone tissue targeting. Further technological advances, such as ligand-dependent affinity or magnetically controlled release, may be necessary to achieve truly selective tissue delivery.

Replacing estradiol therapy with RLX only partially addresses the problem of some side effects. RLX therapy is only associated with a number of new side effects. Therefore, it does not solve the problem; it merely changes their profile (it is oncologically safe, but other serious side effects occur). Therefore, combining it with nanoparticles is justified. It has been shown that the use of nanoparticles in estrogen therapy can be more effective in minimizing its side effects by limiting its action to bone tissue. However, it should be noted that the expected reduction in adverse effects of E2 and RLX combined with nanoparticles is merely a hypothesis that has not been confirmed in any of the articles presented here. Therefore, further research is needed to confirm or refute this hypothesis.

Combining various forms of NP with RLX may also have a positive effect on the treatment of postmenopausal osteoporosis. Slower release and improved bioavailability have been demonstrated, which may contribute to reduced therapeutic doses, thus minimizing the occurrence of side effects [18,19,23,41].

In most nanoparticles, the structures of E2 and RLX were found to be stable. This is important because the pharmaceutical preparation must have good storage stability and a long shelf life. Most combinations also demonstrated reduced cytotoxicity and improved pharmacokinetic parameters of these drugs. Studies involving the combination of NP with E2 and RLX are preclinical. Therefore, further studies are needed to confirm the therapeutic effects of NP in postmenopausal osteoporosis, establish a safe dose, and confirm that side effects are minimized without causing cytotoxic effects in patient cells.

Further studies are needed to confirm the therapeutic effects of NP in postmenopausal osteoporosis treatment, establish a safe dose, and confirm side effect minimization without causing cytotoxic effects in patient cells. One promising approach is the use of nanoparticles, which enables precise drug delivery to target tissues. Such targeting increases estradiol’s therapeutic efficacy while minimizing side effects resulting from its systemic effects. Among the analyzed papers, only the article on PLGA-AL@Fe_3_O_4_/E2 NPs + MF described a targeted effect on bone in vivo [18]. The authors of the analyzed papers hypothesize that the use of nanoparticles with drug delivery may ensure targeted delivery of active substances to specific tissues.

A drawback of these studies is the limited data on targeting and side effects. Furthermore, it should be noted that most of the potential improvements associated with the use of nanoparticles stem primarily from improved pharmacokinetics. Therefore, more research is needed on the mechanisms of active targeting, biodistribution, selectivity, and tissue uptake to better understand these relationships when using nanocarriers.

Encapsulation studies were performed using E2- and RLX-loaded nanoparticles. The results described above showed encapsulation efficiency (EE) values above 90%, which is undoubtedly a very good result [23,25,26]. It should be noted that parameters such as surfactant density, pH, surface modification, and nanocarrier structure increase EE; however, not all presented studies report EE data, indicating the need for further research to develop the most efficient formulations. Among the available articles from recent years, only PLGA-AL@Fe_3_O_4_/E2 NPs + MF and RAL/HSA/PSS NPs have been evaluated for bioavailability [18,27]. Their bioavailability exceeded 2%, which remains a relatively unsatisfactory result.

The hemolysis test is another critical toxicity assessment that must be performed, particularly for injectable nanomaterials [55]. In vitro testing represents a suitable method for the initial evaluation of toxicity [56].

In relative terms, hybrid platforms offer the most favorable balance of encapsulation efficiency, stability, and release control, whereas NLCs and HSAs exhibit greater biocompatibility but lower potential for targeted delivery. A drawback of the presented studies is the use of different animal models and routes of administration, which hampers the assessment of study quality and reproducibility and complicates progression toward clinical trials.

With the promising results from combining nanoparticles with E2 and RLX in the treatment of postmenopausal osteoporosis, their limitations and drawbacks should be carefully considered. There is still a paucity of literature data on their toxicity, physiological barrier bypass, phagocyte evasion, physiological barrier bypass, and immune response generation, among other topics [57].

On the other hand, there are noticeable growth trends in the use of nanoparticles in various fields of medicine [58]. Among the described nanoparticles, lipid polymeric and liposomal nanoparticles seem to have the best prospects, as they were the main categories among newly approved drugs by the FDA [58]. This indicates that the nanoparticles presented in the article could, in the future, after supplementing the data and refining, pass the regulatory barrier and be used in the treatment of postmenopausal osteoporosis. However, despite the large number of preclinical projects, few nanoparticle therapies reach the final stages of clinical trials and obtain approval [59]. It is important to remember that introducing drug nanocarriers into clinical use carries many risks. These include, for example, a lack of regulation, difficulties in standardizing large-scale production, and potential nanotoxicity [59].

Future preclinical studies should adopt standardized reporting frameworks, including mandatory biodistribution analysis in key organs (e.g., liver, spleen, kidneys, lungs, reproductive tissues), standardized cytotoxicity assays on bone-related cell lines (osteoblasts, osteoclasts, MSCs), and evaluation of nanoparticle stability under physiological conditions. Such harmonization would improve study comparability and translational relevance.

## 6. Future Perspectives

It is possible to modify the surface of nanoparticles to target specific cellular receptors. This would undoubtedly increase therapeutic selectivity and reduce the toxicity of certain drugs [48]. Nanoparticles may prove effective in gene therapy, which involves delivering genetic material to target cells to treat or prevent disease [48].

Another challenge in the use of nanoparticles is uptake by the reticuloendothelial system, insufficient targeting, and the formation of pro-oxidants. Therefore, recent research focuses on developing biomimetic drug delivery systems. The goal of these studies is to develop strategies that utilize the body’s natural compounds to overcome biological barriers [59]. In recent years, the prospect of combining the use of nanoparticles with artificial intelligence has also emerged. This can be used to design nanotubes capable of creating scaffolds that contribute to improved drug delivery [60]. This approach offers a real opportunity to increase the effectiveness of therapy while reducing side effects, bringing nanomedicine closer to full clinical application. It is also worth noting that in recent years, mRNA with lipid nanoparticles has shown great therapeutic potential in numerous clinical studies and clinical applications [53]. This method has not yet been used in bone fragility models, but further development of this type of nanoparticle may contribute to the development of better treatment strategies for postmenopausal osteoporosis.

## 7. Conclusions

Nanoparticles represent a promising platform for drug delivery in the treatment of postmenopausal osteoporosis by improving the pharmacokinetic properties of estradiol and raloxifene, including bioavailability, half-life, and controlled drug release. These pharmacokinetic advantages may enable a reduction in the required therapeutic doses without compromising efficacy. Preclinical studies further suggest a potential reduction in toxicity and the frequency of adverse effects, particularly in estradiol-based therapies, which could enhance the safety of long-term treatment. However, current evidence does not provide conclusive proof of active bone-specific targeting, and the observed accumulation is largely attributable to pharmacokinetic and passive distribution effects. Among the investigated systems, lipid-based nanoparticles, particularly liposomes, currently represent the most clinically advanced nanocarrier platform, owing to their FDA approval and favorable biocompatibility profile. Nevertheless, further studies are required to confirm therapeutic efficacy, establish safe dosing regimens, and verify that side effects are minimized without inducing cytotoxic effects in patient cells.

## Figures and Tables

**Table 1:** Comparison of reported side effects associated with systemic E2 and RLX therapy.

Side Effects in Osteoporosis Treatment	Type of Drug	
	E2	RLX
Stroke	+	+
Cardiovascular diseases	+	+
Breast cancer	+	−
Endometrial cancer	+	−
Weight increase	+	−
Venous thromboembolic disorders	+	−
Cholecystitis	+	−
Eye diseases	−	+
Uterine polyps	−	+
Hemorrhages	−	+
Gastrointestinal disorders	−	+

“+” side effect occurs; “−“ no side effect occurs.

**Table 2:** In vivo preclinical studies on the use of nanoparticles in the treatment of osteoporosis.

Type of Medicine	Type of Nanoparticles	Material	Route of Administration	Drug Dose	Pharmacokinetics	Influence on Bones (in Relation to Control)	Zeta [mV]	Encapsulation [%]	Size [nm]	References
E2	PLGA-AL@Fe_3_O_4_/E2 NPs + MF	Female Sprague Dawley rats + in vitro	intravenously in the tail	63 μg/kg	-	↑BV/TV, ↓Tb.N i, ↓Tb.Sp, ↑PINP, ↑OC, ↓CTX, ↓TRAP-5b.	od −4.96 ± 0.51 do −6.35 ± 1.97	58.34	~200	[18]
E2	ZnHA-NPs + E2	Female Sprague Dawley rats	E2 orally, ZnHA-NPs intravenously	20 μg/kg	-	↓RANKL i, ↑OPG, ↑BMD, ↑VEGF, ↑PCNA, ↑PINP, ↓ALP, ↑Ca, ↑P	−19.4 ± 3.94	-	64 ± 10 × 20 ± 3	[19]
RLX	NP RAL/HSA/PSS	Wistar rats + in virto	injection	5 mg/kg	↑Cmax, ↓Vd/F, ↑bioavailability, ↓release into plasma	-	-	-	302.8 (RAL/HSA/PSS-1/2/8) 266.9 (RAL/HSA/PSS-1/2/10)	[27]
RLX	RLX-bNP and RLX-cNP	Sprague Dawley rats + in vitro	through gavage	25 mg/kg	↑bioavailability, ↑Tmax, ↑Cmax, ↑AUC_0–t_, ↑K_el_	-	36.3 and −48.6 (mucin system)	94.47	156	[26]
RLX	RLX-NLC	Female Wistar rats + in vitro	orally	15 mg/kg	↑bioavailability, ↓drug release in the digestive tract	-	14.4 ± 0.5	94.51	120 ± 3	[25]
RLX	RLX-PNP	Sprague Dawley rats	orally	5 mg/kg	↑bioavailability, ↑Tmax, ↑Cmax, ↑AUC_0–∞_	↓ALP, ↓Ca in serum, ↓bone resorption, ↑bone formation, ↓Pyr, ↓Dpyr	24.4 ± 1.2	91.73	134 ± 1.3	[23]
RLX	RLX-NLCs	Female Wistar rats + in vitro	orally	60 mg/kg	↑Tmax, ↑Cmax, ↑AUC_0–72h_, ↑K, ↑Ka, ↑T1/2, ↑MRT	-	−23.6	80.09	186	[43]
RLX	RLX-spanlastic nanogel	Wistar rats + in vitro	transdermally	31 µg	↑bioavailability, ↑Tmax, ↑Cmax, ↑AUC_0–48_ and AUC_0-∞-_, ↑T1/2,	↑BMD, ↑BV/TV, ↓Tb.Sp i, ↑Tb.N, ↑Conn.Dn, ↑Cr, ↓P, ↑E2	-	-	-	[41]
RLX	RAL/VitaD/HSA and RAL/VitaD/HSA/PSS NPs	Female Wistar rats + in vitro	intravenously	5 mg/kg	↑bioavailability, ↓CL/F, ↑Cmax, ↑AUC_0–t_, ↑MRT, ↓Tmax, ↓CL/F, ↓Vd/F.	-	-	-	-	[24]
RLX	L-ORM and RAL	Female Sprague Dawley rats	orally	10 mg/kg	↑bioavailability, ↑Cl, ↑Cmax, ↑AUC_0–last_, ↑AUC_0–∞_, ↑Vd, ↓Tmax, ↑T1/2	-	22.20 ± 0.10	-	94.01 ± 1.79	[40]

“↑”—parameter increase; “↓”—parameter reduction. Pharmacokinetics: Cl—clearance; Cmax—maximum plasma concentration; AUC_0–last_—area under the curve from zero to last; AUC_0–∞_—area under the curve from zero to infinity; AUC_0–t_—area under the plasma concentration versus time curve; AUC_0–72_; AUC_0–48_—magnitudes of model-independent parameters like area under the curve; Vd—apparent volume of distribution; T1/2—half-life; Tmax—time to peak concentration; Vd/F—volume of distribution parameter; K_el_—elimination constant; MRT—mean residence time; K—elimination rate constant; Ka—rate constant; CL/F—clearance parameters. Influence on bones: ALP—alkaline phosphatase; PINP—N-terminal propeptide of type-I procollagen; Pyr—pyridinoline; DPyr—deoxypyridinoline; BMD—bone mineral density; BV/TV—including percent bone volume; Tb.Sp—trabecular separation; Tb.N—trabecular number; Conn.Dn—connectivity density; OPG—osteoprotegerin; RANKL—receptor activator of nuclear factor-kappa B ligand; VEGF—vascular endothelial growth factor; PCNA—proliferating cell nuclear antigen; Cr—creatinine; OC—osteocalcin; CTX-C—telopeptide; TRAP-5b—tartrate-resistant acid phosphatase 5b; Ca—calcium; P—phosphorus.

**Table 3:** The most important advantages of nanomolecules used for preclinical studies on osteoporosis models.

Type of Nanoparticles	Cytotoxicity	Bioavailability Compared to the Control Group	Improvement in Bone Parameters Compared to the Control Group	Targeted Delivery	References
PLGA-AL@Fe_3_O_4_/E2 NPs + MF	No hemolytic activity, cell viability ~90% (MTT test)	no data available	+	+	[18]
ZnHA-NPs + E2	no data available	no data available	+	no data available	[19]
NP Ral/HSA/PSS	no data available	The use of NPs did not result in a reduction in bioavailability.	no data available	no data available	[27]
RLX-bNP and RLX-cNP	no data available	NPs have been shown to improve bioavailability	no data available	no data available	[26]
RLX-NLC	no data available	Better oral bioavailability demonstrated	no data available	no data available	[25]
RLX-PNP	cell viability ~80% (MTT test)	NPs significantly improved drug bioavailability	+	no data available	[23]
RLX-NLCs	no data available	no data available	no data available	no data available	[43]
RLX-spanlastic nanogel	no data available	The results showed improved bioavailability	+	no data available	[41]
Ral/VitaD/HSA and Ral/VitaD/HSA/PSS NPs	No hemolytic activity, cell viability ~90% (MTT test)	Higher bioavailability has been demonstrated	no data available	no data available	[24]
L-ORM and RAL	no data available	Nanoformulation increased the oral bioavailability of the drug	no data available	no data available	[40]

“+”—Improvement in Bone Parameters Compared to the Control Group.

## Data Availability

No new data were created or analyzed in this study. Data sharing is not applicable to this article.
